# Improving patient safety governance and systems through learning from successes and failures: qualitative surveys and interviews with international experts

**DOI:** 10.1093/intqhc/mzad088

**Published:** 2023-10-17

**Authors:** Peter D Hibbert, Sasha Stewart, Louise K Wiles, Jeffrey Braithwaite, William B Runciman, Matthew J W Thomas

**Affiliations:** Australian Institute of Health Innovation, Faculty of Medicine, Health and Human Sciences, Macquarie University, 75 Talavera Rd, Macquarie Park, NSW 2109, Australia; IIMPACT in Health, Allied Health and Human Performance, University of South Australia, GPO Box 2471, Adelaide SA 5001, Australia; Australian Institute of Health Innovation, Faculty of Medicine, Health and Human Sciences, Macquarie University, 75 Talavera Rd, Macquarie Park, NSW 2109, Australia; Australian Institute of Health Innovation, Faculty of Medicine, Health and Human Sciences, Macquarie University, 75 Talavera Rd, Macquarie Park, NSW 2109, Australia; IIMPACT in Health, Allied Health and Human Performance, University of South Australia, GPO Box 2471, Adelaide SA 5001, Australia; Australian Institute of Health Innovation, Faculty of Medicine, Health and Human Sciences, Macquarie University, 75 Talavera Rd, Macquarie Park, NSW 2109, Australia; IIMPACT in Health, Allied Health and Human Performance, University of South Australia, GPO Box 2471, Adelaide SA 5001, Australia; Appleton Institute, School of Health, Medical and Applied Sciences, Central Queensland University, 114-190 Canning Street, Rockhampton, Queensland 4700, Australia

**Keywords:** patient safety, clinical governance, implementation science, quality assurance, health care, health policy, qualitative research

## Abstract

Patient harm is a leading cause of global disease burden with considerable morbidity, mortality, and economic impacts for individuals, families, and wider society. Large bodies of evidence exist for strategies to improve safety and reduce harm. However, it is not clear which patient safety issues are being addressed globally, and which factors are the most (or least) important contributors to patient safety improvements. We aimed to explore the perspectives of international patient safety experts to identify: (1) the nature and range of patient safety issues being addressed, and (2) aspects of patient safety governance and systems that are perceived to provide value (or not) in improving patient outcomes. English-speaking Fellows and Experts of the International Society for Quality in Healthcare participated in a web-based survey and in-depth semistructured interview, discussing their experience in implementing interventions to improve patient safety. Data collection focused on understanding the elements of patient safety governance that influence outcomes. Demographic survey data were analysed descriptively. Qualitative data were coded, analysed thematically (inductive approach), and mapped deductively to the System-Theoretic Accident Model and Processes framework. Findings are presented as themes and a patient safety governance model. The study was approved by the University of South Australia Human Research Ethics Committee. Twenty-seven experts (59% female) participated. Most hailed from Africa (n = 6, 22%), Australasia, and the Middle East (n = 5, 19% each). The majority were employed in hospital settings (n = 23, 85%), and reported blended experience across healthcare improvement (89%), accreditation (76%), organizational operations (64%), and policy (60%). The number and range of patient safety issues within our sample varied widely with 14 topics being addressed. Thematically, 532 textual segments were grouped into 90 codes (n = 44 barriers, n = 46 facilitators) and used to identify and arrange key patient safety governance actors and factors as a ‘system’ within the System-Theoretic Accident Model and Processes framework. Four themes for improved patient safety governance were identified: (1) ‘safety culture’ in healthcare organizations, (2) ‘policies and procedures’ to investigate, implement, and demonstrate impact from patient safety initiatives, (3) ‘supporting staff’ to upskill and share learnings, and (4) ‘patient engagement, experiences, and expectations’. For sustainable patient safety governance, experts highlighted the importance of safety culture in healthcare organizations, national patient safety policies and regulatory standards, continuing education for staff, and meaningful patient engagement approaches. Our proposed ‘patient safety governance model’ provides policymakers and researchers with a framework to develop data-driven patient safety policy.

## Introduction

Avoidable patient harm remains one of the leading causes of global disease burden, despite considerable progress being made, especially in developing nations [1]. In high-income countries, approximately one in ten patients experiences an adverse event [2]. In low- and middle-income countries, it is estimated that 134 million adverse events occur in hospitals per year contributing to 2.6 million deaths annually [2].

An increasing recognition of the importance of patient safety has catalyzed the formation of international and national quality and safety alliances to set priorities and shape policy, and there is now a large body of evidence relating to improvement strategies for reducing harm in health care [3–6]. Recent international efforts include the World Health Organization’s Global Patient Safety Action Plan [2] and the Organization for Economic Co-operation and Development’s safety measurement system [7].

Despite extensive resources and efforts to reduce harm, more evidence is needed in relation to which aspects of patient safety governance provide value in terms of improving outcomes for patients and health systems, versus those that are not effective and therefore waste resources. This study aimed to examine the perspectives of international patient safety experts to identify the [1]: nature and range of patient safety issues being addressed, and [2] aspects of patient safety governance and systems that are perceived to provide value (‘successes’) or are not effective (‘failures’) in improving outcomes for patients.

## Methods

### Study design, sample, and setting

A case study methodology [8] using surveys and interviews was employed and is described according to the Standards for Reporting Qualitative Research Checklist (Supplementary Appendix 1). Case study approaches are appropriate to generate an in-depth, multifaceted understanding of a complex issue such as patient safety interventions and governance in its real-life context [9]. We studied multiple cases simultaneously (a collective case study approach) to enable a broad understanding of the effectiveness of patient safety governance [9, 10]. Experts, Fellows, and Academy members (International Academy of Quality and Safety, IAQS) from the International Society for Quality in Healthcare (ISQua) were made aware of this study via ISQua communication channels (including website notices, newsletters, and email circulations) which directed them to the study website. ISQua is a global professional organization that focuses on facilitating improvements in the quality and safety of health care [11]. At the time of the study, ISQua had 1555 Experts, Fellows, and Academy members, with representation across 83 countries and 6 continents [12]. All participants provided written informed consent prior to study enrolment. Participation was voluntary and no compensation was provided. Participants were assured that no identifiable information would be collected or reported. The study received ethics approval from the University of South Australia Human Research Ethics Committee (no. 203 467).

### Data collection

Data collection occurred between May and September 2021, and comprised internet-based surveys (Microsoft Forms) (Supplementary Appendix 2 and 3) and one-on-one in-depth semistructured interviews (telephone or web-based platform; Zoom) (Supplementary Appendix 4). Two researchers (P.D.H. and S.S.) conducted all interviews; both have patient safety experience, with one (P.D.H.) being an ISQua Fellow. Surveys and interviews sought to explore and understand experts’ experiences about patient safety initiatives in which they were involved. Participants were invited to discuss (via case report or interview) an example of either a patient safety ‘success’, or a persistent problem (‘failure’), and to reflect on the lessons learnt and factors perceived as contributing to improvements or failure to achieve the desired outcomes. Interviews were audio-recorded and transcribed verbatim. All data were anonymized for reporting.

### Data analysis

Survey and interview data were analysed thematically using inductive and deductive approaches [13]. Participants’ perspectives on the key barriers and facilitators to effective patient safety efforts (taken from case study ‘successes’ and ‘failures’) were coded and deductively mapped to the System-Theoretic Accident Model and Processes (STAMP) framework [14]. STAMP is generally applied to accidents or hazard analysis; however, it has been used to analyse systems for governance, for example in road safety [15], electronic medical record safety [16], and risk management in hospitals [17]. It is underpinned by systems and control theory, and considers constraints and controls across system development and operations. STAMP recognizes that in today’s complex world, the causes of accidents are generally nonlinear, inter-related, and represented across multiple layers in the system. Patient safety governance can also be considered as a complex sociotechnical system with a series of hierarchical control structures that impose constraints from high levels to low levels [18]. The resistance and inertia in improving patient safety at scale may be related to its characteristic as a complex sociotechnical system.

The aspects of patient safety governance and systems perceived to provide value or not in improving outcomes for patients were analysed inductively (thematic analysis) [19].

Using the constant comparison method, participants’ perspectives were deductively mapped to the content of the STAMP framework [20–22], and then iteratively refined into a novel overarching systems model with key themes for patient safety governance and improvement. Example quotes from interviewed participants were selected for publication.

### Rigor and trustworthiness

The interview schedule was pilot tested on three non-ISQua members with patient safety experience—their data were not included in the study. All interviews were coded by one researcher (S.S.), with a random subsample (n = 6, 22%) coded independently by a second researcher (L.K.W.). The two researchers met to ensure code consistency, before agreeing on the final coding frame and resultant themes and subthemes. Interview data analysis was undertaken by one researcher (L.K.W.) and verified by another (S.S.). Further discussions were held to confirm saturation across sampling, data collection, and analysis domains for each approach [23]. In the inductive analysis, the researchers ensured that no new codes or themes were being generated and no new theoretical insights gained from the data [23]. For the deductive component, the two researchers ensured that the STAMP framework hierarchical levels were adequately represented in the data (i.e. that all levels within the formulated overarching systems model were sufficiently replete with examples from the data). Interview transcripts and summaries of the analysis were sent to participants to ensure they were satisfied with interpretations of their data, with no suggested amendments received.

## Results

### Study sample

Twenty-seven ISQua participants (n = 16, 59% female) completed the survey and then an interview, representing a response rate of 1.7%. Most hailed from Africa (n = 6, 22%), and Australasia or the Middle East (n = 5, 19% each) and were employed in hospital settings (n = 23, 85%). Participants reported a blend of experience across healthcare improvement (89%), accreditation (76%), organizational operations (64%), and policy (60%). According to participants and illustrated via case study descriptions, the nature and range of patient safety issues being addressed in our sample varied in topic (n = 14), with specific examples of adverse events and patient harm most commonly discussed (n = 5, 38%) (Table 1).

**Table 1.** Participant profile.

Participant profile information	Number of participants (% sample)
Participant sex	
Female	16 (59%)
Male	11 (41%)
Continent	
Africa	6 (22%)
Australasia	5 (19%)
Middle East	5 (19%)
Asia	3 (11%)
Europe	3 (11%)
Indian subcontinent	2 (7%)
North America	2 (7%)
South America	1 (4%)
Highest qualification	
PhD/Doctorate	3 (11%)
Masters	14 (52%)
Postgraduate (unspecified)	3 (11%)
MD (Doctor of Medicine)	4 (15%)
Bachelor	1 (4%)
not reported	2 (7%)
Years of healthcare experience	
0–10	7 (26%)
11–20	7 (26%)
21–30	4 (15%)
> 30	7 (26%)
not reported	2 (7%)
Current primary role and sector*	
*International*	
ISQua Academy Member	1
*National*	
Director, Patient Safety and Quality Centre	2
Accreditation Surveyor	2
Patient Safety Consultant	2
Chief Executive Officer, accreditation agency	1
Clinical Governance Health Screening Program Lead	1
Patient Safety Manager	1
*State-wide/Regional*	
Patient Safety Quality Manager (regional community health network)	2
Director, Capability and Culture (state-wide health service)	1
Chief Quality Officer (state-wide health service)	1
*Healthcare organization (hospital)*	
Patient Safety Quality Manager	4
Medical officer (Anesthetist, Physician, Surgeon)	3
Patient Safety Officer	2
Patient safety advocate	2
Head of Quality Management	1
Clinical Risk Manager	1
*Other roles/organizations*	
Executive Director Patient Safety Risk and Quality (non-profit independent organization)	1
Lecturer (university)	1
Current adjunct roles	
Quality improvement and patient safety officer, hospital	2
Lecturer (university)	1
National Advisor, accreditation organization	1
Surveyor, accreditation organization	1
Patient safety case study or issue discussed	
Specific adverse event or harm (e.g. postpartum haemorrhage, transvalvular percutaneous aortic valve replacement, central line infection)	5
Accreditation standards and assessments—hospital	3
Communication and clinical handover	3
Lack of public/transparent reporting of patient safety issues	2
Formalizing patient safety standards (medication management, infection control)	2
No fault compensation systems	1
National Reporting and Learning Systems	1
National bowel screening ‘listen and learn’ forum	1
Hospital procedure implementation	1
Targeting initiatives at wrong level of management (e.g. CEO rather than frontline)	1
Test result follow-up	1
Workplace culture	1
Patient advocate engagement (e.g. education programs, representation on committees)	1
Personal healthcare system experience (cancer)	1

* participants reported multilevel patient safety experience (% of sample not calculated).

### Synthesis and interpretation

**Figure 1:**
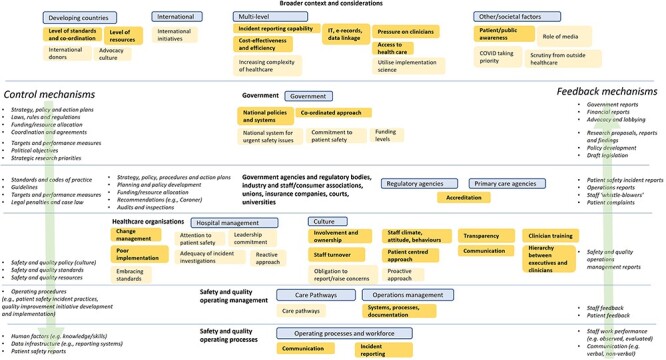
Adapted STAMP systems model for patient safety governance [14]. Note: Control and Feedback mechanisms in italics. Key factors related to patient safety governance and systems are noted in boxes with borders; deeper coloured boxes with bolded text show factors with ten or more responses from participants. These are organised into different STAMP hierarchical levels in plain bold text and sub-levels in boxes with borders. Factors were aggregated including across barriers and facilitators and renamed into neutral factors (for example, lack of access to healthcare (a barrier) and access to healthcare (a facilitator) were aggregated and renamed access to healthcare. Factors mentioned by less than 3 participants were excluded. See Supplementary Appendix 5 for full dataset results.

Thematically, a total of 532 textual segments (n = 247 barriers, n = 285 facilitators) were grouped into 90 factors (n = 44 barriers, n = 46 facilitators) and were used to identify key factors relating to patient safety governance which were aligned with prioritized STAMP framework hierarchical levels [14] (Figure 1). Factors were also used to synthesize four distinct yet interrelated and overlapping themes (n = 12 subthemes) relating to aspects of patient safety governance and systems perceived as valuable or ineffective; these are discussed below and summarized, with illustrative quotes, in Tables 2–4. A matrix of codes, STAMP framework hierarchical levels, and themes appears in Supplementary Appendix 5.

**Table 2.** Theme 1 (organizational culture, safety and quality mindset) summary, illustrated with participant quotations.

Theme 1: organizational culture, safety and quality mindset
Sub-theme: organizational culture, safety and quality mindset
“…A very good patient safety culture, it is an important focus at every level. Leaders set an example by making patient safety the highest priority.” (Participant 13) ‘So, although within that national body bowel screening in Wales, there wasn’t, I wasn’t able to push that forward because the culture, they didn’t understand the importance of it.’ (Participant 20) ‘Patient safety sits in the realm of clinical care, and they’re not experts on clinical care. They are experts on managing a budget, ‘cause that’s what they’ve done historically. So, and that’s just a general statement, I know Boards now have people of the board that have got clinical backgrounds and they have board sub-committees for clinical governance.’ (Participant 1) ‘I think one of the major factors is the leadership on yeah, because of that, have a successful model or they have been successful in improving quality and efficiency.’ (Participant 23) ‘Where we have managers that also have the political background those managers are really involved because they have the time, they have the political power behind them.’ (Participant 11)
Sub-theme: staff engagement
‘Changing a culture, you know, engagement with staff, yeah, routinely..’ (Participant 28) ‘The majority of my colleagues who are specialised in this area, patient safety, the main problem is the culture of safety, the lack of the culture of safety…there is a power distance between the nurses and doctors, between doctors themselves, nurses themselves and the executive team, the Board.’ (Participant 17) ‘Yeah, I think it’s, it’s gotta be bottom up. I think memos from health departments don’t help.’ (Participant 2) ‘It’s important not to dictate, but to empower/champion. Frontline clinicians need to have buy-in.’ (Participant 12) ‘So for me, I think when you involve everybody in that, if you can assess – know where you’re start from, help them in develop strategies and implement them. What you realise is there’s some local ownership of the program. And then they can recommend it the way that would fit their context.’ (Participant 24) ‘To cultivate positive attitudes and behaviours relating to patient safety, organisations should focus on promoting common values.’ (Participant 6)
Sub-theme: endorsement of quality
‘But, you know, what they experience, what they see, you know…the support for them and their wellbeing and having the sort of types of opportunities for reflective practice that enables them just to try and make sense and then stay connected.’ (Participant 8) ‘Yes, yes, yes, we really need to have a culture change. We see very many people who want to cover these errors because they want to retain their hospital name to be one of the best or the best respected in their field.’ (Participant 18) ‘In my organisation they always said they’re not blaming culture or organisations…yes, but I guess in reality, it’s their own perspective that they are afraid to be blamed…’ (Participant 15) “That pre, for the 6–12 months pre-accreditation survey, probably 6 months, yes. All the resources home in on that. And everybody sees it, and so they see, they become even more cynical. Some organisations try to make it ‘this is just the way we do business around here, this is part of business as usual rather than an event’. And that takes a while to shift that culture….So…just because you say that ‘this is business as usual and not an event’, if people see things happening that look like you’re getting ready for an event, then they go: ‘well, I hear what you’re saying but that’s not what I’m seeing…‘They cram for an exam…they leave it to the last minute, cram and then their capacity to keep anything going or to sustain it diminishes significantly after their surveyors leave the organisation.’ (Participant 1) ‘There’s an emphasis in Wales and the UK on a just culture… And just recognising the support that’s needed when people make mistakes…they need to be supported in the same way that the patients or the person at the receiving end of the mistake needs to be supported.’ (Participant 20)

### Theme 1: Organizational culture, safety, and quality mindset

The organizational culture, safety, and quality mindset was the most frequently mentioned barrier or facilitator by participants comprising 412 (or 77% of) textual segments (Supplementary Appendix 5).

### Organizational culture and leadership priorities

Participants were emphatic that a health service organization’s culture is ‘critical’ to setting its safety, quality mindset and efforts. The approaches and activities prioritized by leadership and management teams are indicative of culture and reflect their commitment to safety and quality. These filter through the organization in discussions and meetings with staff (Table 2).

Participants reported that leaders’ priorities are largely shaped by their accountabilities and how success is measured within the organization (e.g. financial key performance indicators versus quality outcomes), and these priorities can either promote or downgrade the importance placed on safety and quality. Safety should be ensconced within the organizational development of health services through adequate clinical representation on Boards, and by employing models of safety improvement with proven track records. The efforts of individual safety managers who can leverage political networks and priorities make a real difference in producing positive outcomes for the community (Table 2).

### Staff engagement

Many participants suggested that safety culture and awareness could be deepened with meaningful staff engagement through partnership in nonhierarchical consultations rather than imposing preconceived solutions, thereby engendering ownership of issues and initiatives. Leaders also need to provide consistent clear messaging and follow-up with actions that align with stated patient safety priorities (Table 2).

### Endorsement of quality

Healthcare employees’ behaviour is guided by decisions from their management teams; therefore, quality improvement initiatives must be endorsed by organizational leaders. If the organization promotes reflective practice and staff-led safety and quality improvement initiatives, a sustainable culture of improvement can grow. On the other hand, factors identified as negatively influencing safety culture included ‘cover up’ activities borne out of concerns for reputational protection and a heavy focus on accreditation compliance as an endpoint rather than business-as-usual. There was a clear consensus that nonpunitive approaches that seek to unearth learnings for future improvement best support patients and staff (Table 2).

### Theme 2: Policies and processes to investigate, implement, and demonstrate impact

Factors related to policies and processes to investigate, implement, and demonstrate impact comprised 68% (361/532) of the textual segments mentioned by participants (Supplementary Appendix 5).

### Policy

National-level reporting and legislative mandates were considered an important drive for organizations to prioritize safety programs and promote transparency in incident reports and learnings (Table 3).

**Table 3.** Theme 2 (policies and processes to investigate, implement, and demonstrate impact) summary, illustrated with participant quotations.

Theme 2: policies and processes to investigate, implement and demonstrate impact
Sub-theme: policy
‘The government publishes reports (publicly) on hospital performance and hospitals are ranked, great incentive to improve. Hospitals are competitive but the focus is not on blame. Do not publish raw data, data compared in a fair way that accounts for differences between hospitals in terms of patients/services. This reporting seems really well accepted by hospitals and health professionals.’ (Participant 13) ‘I think there’s a definite correlation with culture and that comes from policies and national policies. So…in England, I suppose the duty of candour was introduced a number of years ago. And Wales has now done something very similar…whereby there is an emphasis for the NHS to be open and transparent about mistakes or about learning.’ (Participant 20)
Sub-theme: regulatory bodies
‘I am pro accreditation standards. So, I totally believe that….we need to have standards, the same as any industry. We need to have standards that we hold people to account for.’ (Participant 1) ‘It needs lots of improvement, it’s something like first standard of hospital standard accreditation was something like a learning edition. So, we couldn’t do anything that would harm the hospital because we know the hospitals are bad but if you set the bar too high, nobody will get to it and the political interest is to close hospitals, not to develop them.’ (Participant 11) ‘I think the main barrier I foresee is the – the non-empowerment – for lack of a better word, of the regulatory bodies. So, for me the health professional regulatory bodies, I think need to do more towards patient safety I have looked at the standards that our local regulatory body required for patient safety. So, what you see is they have a standard for patient safety that is really minimal really minimal. So, it hasn’t put any demand on the facility to actually drive their agenda.’ (Participant 24)
Sub-theme: processes—data informing and informed by practice
‘We introduced the trigger tool for them, the IHI (Institute for Healthcare Improvement) trigger tool. Yeah. And one of the best organisations in the private sector, they started to use this system to collect the data related to the adverse events, including medication adverse events. So, this, I feel, is a success because this tool helps organisations with the low reporting and the culture of patient safety, they start to have some data about patients.’ (Participant 23) ‘We’ve got resources that are trying to drive patient safety. So, clearly, we identify people that are gonna lead the agenda, lead the conversations. But if all they see, and all they work with is what’s reported in incident report forms, which is pressure ulcers, medications and falls, then that’s where their energy goes. Rather than looking more broadly and saying those questions: what procedures are we doing here where the evidence is that it’s low value, you know? That perhaps, you know, from morbidity mortality we shouldn’t have operated, how are we supporting people to make the right decisions about the care we provide and the quality of that care? And whether it’s appropriate?’ (Participant 1)
Sub-theme: implementation, interpretation, and demonstrating impact
‘Sometimes the standards may be given out at the national level, but they’re just not translated on the ground. They just remain as things in the document, but there’s no follow up to really make sure that they’re implemented at the facility level.’ (Participant 9) ‘Which ones can we tick off first that say we’ve actually implemented that recommendation? So, I think, depending on what the issue is, and depending how high-profile it is in the media, ‘cause I think the media play a huge role in pushing the health profession to change. So, if it ends up front page of the paper then it can provide a trigger for change.’ (Participant 1)

### Regulatory bodies

Regulation agencies and accreditation processes were considered key to ensuring accountability, particularly when underpinned by government support and industry-based partnerships. National-level standards were viewed favourably; however, potential negative consequences of ‘setting the accreditation bar too high’ included the closure rather than development and improvement of ‘problematic’ health services. Other potential limitations of accreditation included setting patient safety standards too low, inadequate assessment, and failure to partner with health services to undertake the necessary improvements (Table 3).

### Processes—data informing and informed by practice

Many participants described the value of high-quality data systems such as incident reporting and trigger tools that align with key safety priorities. It was noted, however, that incident reporting systems do not provide the complete picture. Nontraditional data sources should be used to validate and enhance information from reporting systems and identify issues that are not routinely reported (e.g. use of low value procedures, rates of and time to patient follow-up care). Data ‘drill-downs’ should be undertaken by dedicated quality and safety managers, in conjunction with staff discussions, to flesh out the underlying reasons for ‘why’ and ‘how’ things go wrong. Engagement by healthcare professionals is often low due to a failure of organizations to ‘close the loop’ in response to identified issues, coupled with the burden of completing often lengthy incident forms (Table 3).

### Implementation, interpretation, and demonstrating impact

Participants espoused the value of implementation approaches that ‘start somewhere’ and are ‘clinically relevant’, such as risk stratification and pain score assessments on hospital presentation. Contrarily, strategies undertaken at the level of paperwork (e.g. hospital procedural documents) are sometimes viewed as not resulting in meaningful improvements and done ‘for show’ (Table 3).

Some examples of limitations to demonstrating impact from initiatives included inadequate assessment of safety issues, rolling out off-the-shelf solutions rather than tailored context-sensitive approaches, inappropriate selection of outcome measures (i.e. flawed unidimensional short-range metrics), and too much focus on either celebrating improvements or calling out residual deficiencies (e.g. acceptability thresholds for hand hygiene rates). Political imperatives can lead to patient safety issues ‘of the moment’ gaining attention at the expense of others, such as increased health service activity or ambulance queuing, or hospital readmission rates (Table 3).

### Theme 3: Staff support, upskilling, and shared learning

Factors related to staff support, upskilling, and shared learning comprised 68% (374/532) of the textual segments mentioned by participants (Supplementary Appendix 5).

### Staff support and capacity

A supportive organizational ethos that focuses on learning rather than blame will develop staff as agents of patient safety change. Staff can then feel comfortable to report issues, fostering positive reporting practices within the organization. Staff in safety and quality roles needs to be supported with sufficient resourcing to ensure a critical mass of field teams and managers, and allowed license to balance their safety responsibilities with other clinical pressures (Table 4).

**Table 4.** Theme 3 (staff support, upskilling, and shared learning) and Theme 4 (Patient engagement, experiences, and expectations) summary, illustrated with participant quotations.

Theme 3: staff support, upskilling, and shared learning
Sub-theme: staff support and capacity
‘So, like my early examples where you know, that culture just didn’t support a reporting or a learning organisation. I think if they [staff] feel supported and comfortable that you know, nothing adverse will come, it’s just about learning, I think they’re more than happy to support that process.’ (Participant 20) ‘Usually the nurses are mostly involved in quality management. But in big hospitals they’re really stressed because they have the doctors, the residents, that put pressure on them, we put pressure on them. And in hospitals there are really few, they don’t have enough staff, so there’s another type of pressure.. I think the solution is to, to get, get more in the field.’ (Participant 11) ‘I think the most challenging is about, I suppose, getting everyone onto the same value proposition that it’s a good use of their time. Obviously, everyone’s always busy and everyone’s got priorities and actually, you know, getting, having, sort of, different conversations with different people to actually find a thing that connected to say this is a good use of your time. Because you need that buy-in, that sort of buy-in. There are a lot of willing people but they’re willing to do it in their own time. But if you actually want to do this together, you’ve got to, there’s a compromise that, you know, has to happen from there. So I think that’s probably, and that takes time. And, you know, it’s all about agile and, you know, quick movements and parts from there. But if you don’t spend that, you know, you do have to invest time to make time.’ (Participant 8) ‘The characteristics of effective initiatives include empowerment of staff and having safety/quality Champions.’ (Participant 9)
Sub-theme: staff upskilling
‘I think we have a big problem on education because patient safety, quality management nobody teaches it in the faculty of medicine or the residency.’ (Participant 11) ‘Clinician education in safety/quality plays an important role. Education needs to be ongoing through the career.’ (Participant 9) ‘We also are teaching hospital, and you see most of the health care workers wherever they are, we do also teach some of these models of patient safety, so that right, when they go back to their facilities, then well, they their workforce can actually start implementing these.’ (Participant 18) ‘Or that like, for example, university hospital staffers and the big private hospitals they have well-established, say, residency programs for the physicians and they are an educational hospital. And they are working a lot on building capacity of their health care providers, including physicians, nurses and other health care providers.’ (Participant 23) ‘Really think that if we could push, that we have these discussions right from medical school, nursing school it will really make a big impact. It will change the attitudes of the professionals, even as they come to practice. So, that is one of the biggest gaps that exist. When we come to talk to these people and they have already been in the system, they have already developed bad cultures, so again, changing those attitudes will take a bit of time.’ (Participant 18) ‘I think there’s an awful lot that needs to go into teamwork and the non-technical skills, the communication skills for everybody.’ (Participant 20) ‘So, we address processes to say: “Let’s put in place huddles” and “let’s put in place improved ways for people to hand over” and “let’s talk about open disclosure”. But we don’t address the fact that we have a huge population of people working in health that actually don’t know how to communicate well, that don’t know how to have a good conversation, that don’t listen. So, we’re not dealing with the human factors.You can mandate a huddle and you can mandate handover, mandate all of these sorts of things but when you talk to people about communication in health, often it’s the attitude, behaviour of individuals and their communication skills.’ (Participant 1)
Sub-theme: hared learning
‘We have a, like ISQua webinars, like an hour. We call them the “quality hour”. We present best practices from our country. Hospitals that really stood up and how did they do that. And it’s interesting because we have seen something like 400–500 people watching every time. Mostly doctors, managers from, from the systems. Things are getting to others so the information is flowing.’ (Participant 11). ‘International collaborations have been valuable – learning what has been successful in other countries.’ (Participant 16)
Theme 4: patient engagement, experiences, and expectations
Sub-theme: patient engagement and experiences
‘When we come to talk to these people and they have already been in the system, they have already developed bad cultures, so again, changing those attitudes will take a bit of time, like I said I’ve been here for 25 years, and they have never heard about patient centred issues.’ (Participant 18) ‘Emerging Leaders in Patient Safety, we had on the, we had 30 scholars, 10 faculty, 2 or 3 of the faculty were always patient advocates. And the feedback from the scholars saying: this is the best part; I’d never dreamt of the patient’s experience before. And several of those people totally changed their careers and gone into patient safety as a result of the, you know.so, you know, I think the patient experience is something people need to hear.’ (Participant 2)
Sub-theme: patient expectations
‘Patient attitudes and health literacy beginning to show signs of change but will take a long time to alter entrenched culture of healthcare.’ (Participant 5) ‘We have the problem of the patients, like, the education level is really low. There is not a complaint culture in Romania. They say if you have a problem, the hospital or anywhere else, you’re just, OK, if you manage this and nothing really bad happened, you won’t do a formal complaint or something. So, this is a problem ‘cause there are a lot of risks and a lot of adverse events happen in the hospital but the patients do not follow them through. So, if they, let’s say, if a patient falls on the stairs because of the medication, and he stays a few days more in the hospital, let’s say he doesn’t get a bad injury. He says: “Thank God it wasn’t worse.” And that’s all.’ (Participant 11) ‘That is part of what I’m doing presently continuously to engage and advocate and … all the patients they … practitioners in the health care system, that’s the communication and collaborative is not … they are … doctors believe they are the top … so they notice … tend to be afraid or there is conflicts in working together and what they want … is to ensure that is collaborative and adequate … communication, similar the health care staff and this will also work for the patients themselves for them to know that they are not passenger, they are partner in their own care. So, if patients know their rights to be able to inform the doctors or nurse or whatever in this system that this is what is … for, like if you have health care accreditation … be able to speak … the doctor … procedure … to me, so this how was me trying to improve on, so presently there are communication challenges within the health care.’ (Participant 21)
‘Well…the health systems are different; not because of what they do but how they manage their culture at a broader level. So, in China, of course, huge populations, they don’t have the same requirements around volumes of staff versus numbers of patients. So, the clinicians there deal with significantly more patient load than what clinicians here would deal with. But of course, here, outside of safety and quality you’ve also got unions that have a voice. And you’ve got public discussion around number of hospital beds, number of nurses, the number of doctors, so…you’ve got much more open conversation about, and more open opportunities to criticize and have your politicians respond that you do in other countries that are…Yeah, yeah. ‘Cause it’s public pressure. Whereas that’s emerging in places like China, that’s emerging, that public pressure and that public voice. But it’s still, there is still, I think, an umbrella of silence. People don’t want to be critical and they are, like I said, they’re dealing with extremely large numbers of….I mean, that’s, the clinicians here would just not cope, not cope at all with the volume. So, their approaches to safety and quality have to be different because of culture, volume, the fact that they’re not, they don’t have that union voice, you know, all those other sorts of things that impact on health system as part of a broader system of public services.’ (Participant 1)

### Staff upskilling

Healthcare workers often lack safety-specific technical knowledge and skills to adequately understand and drive improvement agendas within their organizations. Thus, career-long safety education should be made available. Responsibility for continuous learning falls within the remit of both university and teaching hospital sectors and is reflective of an organization’s commitment to building capacity for safety. Practical and experiential learning opportunities are especially valuable in helping staff to appreciate the nature and range of patient safety problems (Table 4).

Education on foundational topics is also essential and should encompass the influence of social determinants of health to enhance understanding of responsible management of health funding and use of cost-effectiveness measures. Human factors were also highlighted as a key educational target. Shaping helpful staff attitudes, behaviours, and communication skills is critical to these being perpetuated within healthcare organizations (Table 4).

### Shared learning

To achieve ongoing improvement in the patient safety space, there must be opportunities for all stakeholders to share information and learnings which tend to increase buy-in from all sides. Webinars and other virtual modalities were suggested as a means of communication across a country (Table 4).

### Theme 4: Patient engagement, experience, and expectations

Factors related to patient engagement, experience, and expectations comprised 22% (118/532) of the textual segments mentioned by participants (Supplementary Appendix 5).

### Patient engagement and experience

There was widespread recognition that patient engagement is pivotal to patient safety. However, in some regions, patient-centeredness is still a relatively new concept that is not embedded in organizational policies, education, and practice. Nevertheless, there was general agreement that hearing patients’ personal stories positively influences healthcare professionals’ attitudes towards patient safety, with some subsequently attracted to patient safety roles. Leadership training, in particular, could benefit from incorporating ‘patient experience’ first-hand from patient advocates (Table 4).

### Patient expectations

Patients’ health literacy influences their own expectations and attitudes towards safety. Where levels of health literacy are low, it was reported that some patients almost ‘expect and accept’ adverse events. Conversely, participants reflected that well-informed patients are active partners in their own care, and can advocate for their own safety especially when things go wrong. Public education, implemented early and with due sensitivity of cultural nuances, may enhance patients’ self-efficacy through a heightened understanding of basic healthcare principles. Collaborative person-centred approaches and general improvements in health literacy can help drive public pressure and expectations of the healthcare system, and organizations must keep pace with these developments.

## Discussion

### Statement of principal findings

The nature and range of patient safety issues being addressed in our sample varied in topic (n = 14), with specific examples of adverse events and patient harm, relating to medical procedures (e.g. postpartum haemorrhage, transvalvular percutaneous aortic valve replacement, and central line infection) most commonly discussed (n = 5, 38%). Four themes for improving patient safety emerged from this study [1]: an organizational culture that prioritizes patient safety [2]; policies and processes to investigate patient safety issues, and implement and demonstrate impact from initiatives [3]; supporting staff to foster appetite, capacity, and participation in safety efforts, and offering upskilling and shared learning opportunities; and [4] engaging patients to explore and enhance their experiences and expectations of safe health care. Barriers and facilitators, and the strategies identified for addressing them, were related to the crafted themes and interconnected within a broader patient safety system.

While these four themes may appear particularly familiar in the context of the patient safety agenda over the past few decades, the results of this study underpin that these areas remain a priority. Further, given the relative lack of new emergent themes, the broader results of the study could be interpreted from the perspective that our recent efforts in these areas have not been sufficient, especially in the face of the many barriers identified by the experts in this study.

### Strengths and limitations

Case design methodology is suitable to answer questions of ‘how’ and ‘why’ in a real-life context [8]. Our sample size of 27 experts was appropriate to explore study aims and achieve data saturation, within pragmatic considerations [24]. The response rate (1.7%) was low but consistent with expectations for a large-scale email circulation approach [25]. Our findings are a function of the convenience sampling approach used. A key study strength was the international perspectives captured through ISQua members, and the triangulation of contexts across countries, health services, and policy settings [26]. Data collection sought case study examples of aspects underpinning high-value and/or ineffective patient safety governance; however, participants’ responses varied in detail. To enhance credibility, we double-coded data from a 20% participant subsample and used a combined deductive-inductive analytical approach. Our findings are a reflection of the STAMP framework chosen for analysis; additional or alternative groupings of critical patient safety governance and system factors may have surfaced from deductive mapping to other frameworks, especially those that centre around or more broadly represent ‘culture’, which comprised 77% of mentioned factors by our participants. Data collection occurred in English; for some experts, this was not their first language which could introduce bias. To mitigate this, we cross-checked interview transcriptions against the audio files to correct for language mistakes. Study participants worked mostly in hospital settings and developing countries; therefore, it is unclear how experts from other settings experience patient safety and how this might affect future studies and recommendations.

### Interpretation within the context of the wider literature

When examined as a whole, common to each of the themes is a focus on the primary actors in patient safety: the patient; the healthcare workforce; and the organization. The Institute for Healthcare Improvement’s framework for respectful management of serious clinical adverse events first emphasised these layers over a decade-ago [27], and the findings of our research emphasize that globally our work needs to continue to develop across each of these ‘layers’ a culture that underpins safety and quality.

Reflecting the outermost layer of the ‘organization’, the study’s most frequently mentioned barrier or facilitator, ‘organizational culture and safety and quality mindset’, features prominently in the well-documented ‘third age in safety’ [28]. Here, the focus is on trying to understand and strengthen everyday features of work within complex sociotechnological systems that promote safety [29]. The achievement of a ‘restorative just culture’ has become an important component of this. Its function is to fashion appropriate responses to evidence of errors and failures and to preserve the possibility of learning while holding people accountable for unacceptable behaviours [29]. Therefore, our study’s key contribution to the literature lies in the findings embodying restorative just culture principles [30], and operationalizing these within a patient safety governance system [31]. Restorative responses to patient safety incidents are ‘a voluntary, relational process where all those affected by an adverse event come together in a safe and supportive environment, with the help of skilled facilitators, to speak openly about what happened, to understand the human impacts, and to clarify responsibility for the actions required for healing and learning’ [32]. This definition speaks directly to the themes identified by study participants, with our findings further supported by a recent systematic review [33] which framed trust as a key tenet to just culture in hospital settings, and achievable through strategies that address organizational and team factors (e.g. management support and commitment, transparent incident reporting, close supervisory relationships, discussion of the nature of errors) and staff experience (e.g. clinical skill confidence, knowledge of reporting systems) [33]. Mirroring related literature, restorative approaches are scaffolded by adequate resourcing [31] and accreditation processes within a ‘quality-of-care (accreditation, inspection and public reporting)’ [34] paradigm. In this way, data-driven solutions can be formulated to ‘close the loop’ and facilitate sustained learning, implementation, and evaluation of patient safety initiatives [35, 36].

With respect to the ‘layer’ reflecting the healthcare workforce, healthcare professionals are often the ‘second victims’ of patient safety incidents, with many personally traumatized and professionally (e.g. blamed) as a result [37]. Continuing the focus on the need for continued efforts to support a culture of safety, the theme of supporting staff in nonpunitive pro-learning environments has been reported in the literature and can assist their wellbeing and motivate reflective practice and behavioural change [37–39]. Support resources for those affected by patient safety incidents aim to provide psychological first aid, foster coping strategies, and promote individual resilience [40]. The Institute for Healthcare Improvement’s framework for respectful management of serious clinical adverse events [27] addresses the second victim phenomenon and has been adopted within national-level patient safety responses [41].

At the core of health care is the ‘layer’ that represents those that receive care and are consumers of our health services, and again, this was a clear theme within the findings of this study. There is increasing global momentum in promoting the patient voice in patient safety initiatives [42]. Recent systematic review evidence supports the benefits of patient engagement when developing health services and policy, resulting in positive health outcomes (e.g. reduced neonatal mortality) and the identification of broader healthcare priorities (e.g. environmental, educational, employment conditions) [43]. In support of our findings, barriers to patient safety engagement are currently based on an unwillingness to participate from patients (e.g. fear of reprisals, health literacy) and healthcare professionals (e.g. potential legal ramifications, attitude/knowledge limitations), coupled with organizational constraints (e.g. power-dynamic cultures, limited resourcing) [43]. Based on available evidence [44], future engagement approaches should be proactive and employ collaborative patient-professional partnerships, user-friendly patient safety feedback systems (e.g. succinct, plain language, integrated with documentation systems), and strategies to empower patients and improve confidence and seek organizational sponsorship (e.g. transparent value-based culture, staff consistency/training, whole of agency support).

### Implications for policy, practice, and research

Policymakers can use our findings to identify and target current gaps in patient safety directives with national and international improvement initiatives that are tailored to a country’s own stage of evolution. These include accreditation processes, consistent and continuous undergraduate and postgraduate education, data reporting systems and infrastructure for benchmarking, and the establishment of clinical collaboratives for shared learnings and work towards national and international priorities. Study themes could inform further explorations of key factors for effective patient safety governance via implementation mechanistic and logic modelling [45], with findings used to design and pilot test interventional approaches across healthcare organizations and settings to explore outcomes and the relative cost-effectiveness in varying contexts.

## Conclusion

Exploring the perspectives of international experts towards patient safety governance revealed aspects that provide value in improving patient outcomes. The quality and sustainability of patient safety governance relies on a strong safety culture in healthcare organizations, national policies and regulatory standards, continuing education for staff, and meaningful patient engagement approaches. The proposed ‘patient safety governance model’ provides policymakers and researchers with a framework to develop data-driven policy and initiatives.

## Supplementary Material

mzad088_Supp

## Data Availability

The dataset generated and analysed during the current study is not publicly available.
