# Supplementary material for: Improving patient safety governance and systems through learning from successes and failures: qualitative surveys and interviews with international experts
Source: Int J Qual Health Care. 2023 Oct 17;35(4):0. doi: 10.1093/intqhc/mzad088 (PMC10656601; doi:10.1093/intqhc/mzad088)
Supplement: mzad088_Supp [file mzad088_supp.zip › Supplementary material_Appendices 2-4_Patient Safety project.docx]

**Supplementary Material.**

**Appendix 2. *Demographic survey.***

**1. Name: __________________________________________________________________________________**

**2. Gender:** Male / Female **3. Country of residence:** _________________________

**4. Highest qualification: ____________________ 5. Occupation:** _________________________________

**6. In which of the following areas of the health system do you have professional experience? *(Tick all that apply)***

| **Role** | **Healthcare level** |
| --- | --- |
| - Accreditation – health service or health professionals | - Primary care |
| - Development of standards – national or regional | - Hospital care |
| - Regulation & compliance | - Other____________________________________ |
| - Health service organisational governance | - N/A |
| - Research |  |
| - Policy development |  |
| - Health service operational |  |
| - Healthcare improvement |  |
| - Clinical |  |
| - Other___________________________ |  |

**7. How many years have you worked in the health system? ____** years

**Appendix 3. *Case study survey questions*.**

**Instructions:**

1. Please respond to either:
   1. Question 1 (a patient safety problem that has been successfully addressed), or
   2. Question 2 (a patient safety problem that has not been adequately addressed).
2. In providing your response, please reflect and report on your own professional experience(s).
3. You are welcome to provide additional documentation (e.g. Word or PDF file formats, PowerPoint slides) to support and provide context to your responses, if you choose (not a required field).

**Question 1**

**Please consider a patient safety problem in your country or area of expertise that has been successfully addressed and led to improved patient outcomes.**

1. **Describe the patient safety issue. Why was it an important priority?**
2. **Describe the solution(s).**

- *Consider all levels and elements of patient safety that may have contributed to the outcome, including national/state (regulation, accreditation, standards), organisational and clinical.*
- *Describe both the type of initiative and the characteristics that made it successful.*
- *Which personnel were involved in implementation, e.g. managers, clinicians, technical experts?*
- *What were the challenges and how were they overcome?*

1. **How was it funded and would you consider it cost-effective?**
2. **Describe the outcome(s).**

*For example: reducing the rate of hospital-acquired infections.*

*If possible, describe how the outcome was measured. Examples could include:*

- *Hospital data on prevalence or rates relating to a cause of death, diagnosis etc.*
- *Patient reported outcome measures*
- *Reduction in waiting times (e.g. elective surgery).*

1. **Was the successful outcome sustainable? Why / why not?**

**Question 2**

**Please consider a persistent patient safety problem in your country or area of expertise that has not been adequately addressed.**

1. **Describe the patient safety issue and why it is an important priority.**
2. **What approaches have been taken to date to address the problem and why, in your opinion, have they been ineffective?**

*Consider all levels and elements of patient safety that are relevant, including national/state (regulation, accreditation, standards), organisational and clinical.*

*Which of these factors do you think is responsible for the problem persisting?*

- *Lack of attention to the problem*
- *The wrong type of approach to address the problem*
- *Problems with implementation*
- *Other barriers e.g. resource constraints, cultural factors*

1. **As a first priority, what type of initiative do you think would be most effective in addressing the problem, considering cost-effectiveness and any constraints that you have identified?**

**Appendix 4. *Interview schedule*.**

**Introduction:**

- *Welcome / what we are trying to achieve*

Thank you for agreeing to do this interview. As you know we are examining patient safety arrangements in different countries to increase our understanding of which measures are most and least effective. We hope that this will help to inform patient safety priorities so that resources can be used most effectively to achieve the best outcomes for patients.

- *Honesty / audio taping*

It is very important that we get your honest opinions and remember that everything discussed in this interview and your specific opinions will remain completely anonymous. We will be making an audio recording of this session for transcription purposes, however only the research team will have access to these recordings. You will not be individually identified in any of our presentations or publications.

**Interview Questions:**

1. How would you describe your role in patient safety? Is your focus at the international, national, state, organisational level – or a combination?
2. Do you think that patient safety has improved over the last ten years? If so, what have been the key drivers?
3. Are you optimistic that patient safety will improve over the next ten years? Why or why not?
4. What do you think are the primary factors that either help you or prevent you from being able to improve patient safety in your role? (barriers/facilitators)

In the next part of the interview, please reflect on your own professional experience in relation to either:

1. a patient safety success, OR
2. a persistent patient safety problem

Option 1: A patient safety success

Please consider a patient safety problem in your country or area of expertise that has been successfully addressed and led to improved patient outcomes.

1. Could you please describe the patient safety issue. Why was it an important priority?
2. Could you please describe the solution – both the type of initiative and the characteristics that made it successful.
3. Which levels and elements of patient safety do you think contributed to the outcome – for example national or state such as regulation, accreditation or standards, organisational or clinical?
4. Which personnel were involved in implementation, e.g. managers, clinicians, technical experts?
5. What were the challenges and how were they overcome?
6. How was it funded, and would you consider it cost-effective?
7. Could you please describe the outcome(s) from the patient’s perspective. For example, reducing the rate of hospital-acquired infections.
8. Was the successful outcome sustainable? Why / why not?

Option 2: A persistent safety problem

Please consider a persistent patient safety problem in your country or area of expertise that has not been adequately addressed.

1. Could you please describe the patient safety issue and why it is an important priority.
2. What approaches have been taken to date to address the problem and why, in your opinion, have they been ineffective?
3. Which of these factors do you think is responsible for the problem persisting and why?

- Lack of attention to the problem?
- The wrong type of approach to address the problem?
- Problems with implementation
- Other barriers e.g. resource constraints, cultural factors

1. As a first priority, what type of initiative do you think would be most effective in addressing the problem, considering cost-effectiveness and any constraints that you have identified?

**Closing comments**

Please tell us about anything else you feel is important for us to know.

Thank you for your time.

**End of interview**
